# miR-21, miR-93, miR-191, miR-let-7b, and miR-499 Expression Level in Plasma and Cerebrospinal Fluid in Patients with Prolonged Disorders of Consciousness

**DOI:** 10.3390/neurolint15010004

**Published:** 2022-12-29

**Authors:** Tatiana A. Petrova, Sergey A. Kondratyev, Anna A. Kostareva, Roman V. Rutkovskiy, Irina A. Savvina, Ekaterina A. Kondratyeva

**Affiliations:** 1 Almazov National Medical Research Centre, Institute of Molecular Biology and Genetics, 197341 St. Petersburg, Russia; 2 Almazov National Medical Research Centre, Polenov Neurosurgical Institute, 191014 St. Petersburg, Russia; 3 Almazov National Medical Research Centre, Anesthesiology and Intensive Care Department #12, 197341 St. Petersburg, Russia

**Keywords:** miR, prolonged disorders of consciousness, vegetative state/unresponsive wakefulness syndrome, minimally consciousness state, traumatic brain injury, hypoxia, polymerase chain reaction, biomarker

## Abstract

In recent decades, significant progress has been achieved in understanding the mechanisms of disturbance and restoration of consciousness in patients after severe brain damage resulting in prolonged disorders of consciousness (pDOC). MicroRNAs (miRs) may be potential candidates as possible biomarkers for the classification of disease subtypes, and prognosis in patients with pDOC. The aim of the study was to analyze miRs expression levels (hsa-miR-21-5p, hsa-miR-93-5p, hsa-miR-191-5p, mmu-miR-499-5p, hsa-let-7b-5p) by a real-time polymerase chain reaction in plasma and cerebrospinal fluid (CSF) from patients with pDOC and to identify a potential biomarker for dividing patients into groups according to disease severity. We analyzed the levels of investigated miRs in pDOC patients, divided by etiology, CRSI, and the total group compared with controls. Our results showed that dividing patients with pDOC into groups according to the etiology of the disease resulted in the most significant differences in the levels of miR-93, -21, and -191 in CSF and plasma samples between groups of patients. Among the analyzed miRs, we did not find a marker that would help to distinguish VS/UWS patient groups from MCS. Examining of miRs as possible prognostic markers in patients with pDOC, the starting point seems to be the cause that led to the development of the disease.

## 1. Introduction

In recent decades, significant progress has been achieved in understanding the mechanisms of disturbance and restoration of consciousness in patients after various variants of severe brain damage. Severe brain damage includes traumatic brain injury (TBI), subarachnoid hemorrhage (SAH) and hypoxia resulting in prolonged disorders of consciousness (pDOC). pDOC are divided into: vegetative state/unresponsive wakefulness syndrome (VS/UWS), where patients have reflexive responses but no sign of awareness, and minimally conscious state (MCS), in which patients show unstable conscious behavior but cannot communicate or intentionally use objects [1,2]. MCS patients were subcategorized into two subgroups: “MCS minus” (MCS−) and “MCS plus” (MCS+). MCS− patients showed low-level purposeful behavior without command following (e.g., visual pursuit, localization to noxious stimulation, object localization (reaching), automatic motor response, and appropriate smiling or crying related to external stimuli). MCS+ patients presented higher-level behavioral interactions (e.g., a movement in response to a command, nonfunctioning communication, and intelligible verbalization) [3,4].

Studies using neuroimaging and neurophysiological techniques have shown that in the group of patients with pDOC, stable repetitive structural changes that uniquely correlate with modern ideas about the morphological substrates of consciousness were not observed [4,5,6]. Functional disorders are also heterogeneous. For example, in 15–20% of pDOC patients, the phenomenon of cognitive-motor dissociation was revealed, when in the absence of obvious behavioral reactions, during additional studies, the level of induced brain activity was revealed, indicating the presence of the so-called “covert” consciousness [7,8]. To develop effective algorithms for outcome prediction, as well as targeted methods or treating pDOC patients, it is important to study the phenomenon of pDOC at various levels, including microstructural. 

Traditional studies of biochemical processes accompanying various variants of brain damage reported specific changes for ischemia, hypoxia, inflammation, and combined variants of brain damage which allowed the development of methods for their corrections. However, the reliable connection between those changes and prolonged disorders of consciousness was not found. Research of various neuromediator levels in serum and cerebrospinal fluid for this category of patients is limited by alterations in the reactivity of receptors for which these mediators serve as ligands [9,10,11]. As a consequence, minimal amounts of any mediator can cause hyperergic reaction; in contrast, increased concentrations, are not accompanied by any responses. In our opinion, at the level of intracellular integration, the study of “batch” stable variants of biochemical processes programmed at the gene level is promising for pDOC patients.

Luppi and colleagues outlined the following main direction in the field of pDOC within the framework of integrated translational science: research of the interrelationships of the “structure and functions” of the brain, analysis of the phenomenon of disorder and restoration of consciousness at the micro and macro levels [12]. Authors suggest that, at present, macroscopic data obtained using neuroimaging techniques should be integrated with the results of studies at the cellular, molecular, and genetic levels, since the subcellular level has a greater prospect for identifying genetic and molecular markers of the clinical course and outcome of pDOC. Specific genetic factors associated with pDOC have not been identified at the moment, but genetic risk factors for adverse outcomes in TBI and hypoxia are known [13]. In this regard, miRs could be potential candidates as possible biomarkers for the classification of the disease subtypes, and prognosis in patients with pDOC, monitoring response to medications, and tracking disease progression. 

MiRs are highly conserved, small (18–22 nucleotides) non-coding RNAs that post-transcriptionally regulate gene expression by suppressing the translation of their complementary messenger RNAs. They play an important role in many biological processes, including cell cycle regulation, cell differentiation, apoptosis, metabolism, and many others [14,15]. These molecules are present in all biological fluids and body tissues and have tissue- and developmental-stage-specific patterns. It is known that miR expression levels can change rapidly depending on the presence of a disease and the functional state of the organism. These facts make miRs potential early biomarkers for various diseases and possible targets for treatment. In this regard, the expression profiles of various miRs in normal and pathological conditions are currently being widely studied [16,17,18]. 

It is known by now that the brain contains a diverse set of miRs [19]. The fact that levels of miR expression vary between different intra- and extracellular structures (neurons, axons, neuromuscular junctions, etc.) may indicate that certain miRs are involved in the regulation of plenty local process providing neuro impulses transmission, implementation of maturation processes, brain development, formation of neural networks, neuroplasticity, and other. In this connection, a large number of scientific papers are devoted to the study of miRs in patients with various neurological and psychiatric diseases, which show significant deviations in the expression of various types of miRs in patients with neurodegenerative diseases, various phases of psychiatric diseases, and traumatic, anoxic brain damage [19]. 

TBI, SAH, and hypoxia are known to be major contributors to pDOC. There is a large amount of data about miR levels in patients after traumatic events [20,21]. The acute phase of TBI is accompanied by a large number of systemic and local reactions of CNS, it is extremely difficult to assess the certain level of miR expression in rapidly changing conditions. One of the first studies of miR expression in a small group of patients with TBI showed a change in miR-671-5p. Other authors showed that miR-9 and miR-451 were differently expressed when measuring in CFS of 11 TBI patients [22]. Subsequent studies included large groups of patients with TBI showed that increased levels of miR-93, miR-191, and miR-499 in acute period correlated with severity of TBI [23]. As the technique improved, the number of miRs studied increased. In 2016, the study of 740 miRs was performed in patients with TBI, of which miR-126-3p and miR-3610 were assessed as prognostically significant [24]. The next stage of development was the study of miRs in dynamics, but no certain data could be used as a reliable diagnostic and prognostic tool. One study showed that miR-142-3p and miR-423-3p increased risk of post-concussive syndrome after TBI, results of other study assumed low level of miR-425-5p after TBI and correlation with the period of TBI [17,25]. A review by Martinez and Peplow summarized data on all known miRs from plasma and CSF that may serve as potentially valuable indicators of diagnosis, severity, and prognosis of TBI [26]. In particular, in the research of Redell and colleagues on the analysis of plasma miR expression in patients with severe TBI compared to healthy volunteers, plasma levels of miR-765 were shown to be significantly elevated, while miR-16 and miR-92a levels were significantly reduced. In contrast, plasma levels of miR-16 and miR-92a were increased in patients with mild TBI [27]. MiR 423-3p has shown potential clinical significance for highlight a group of patients after severe TBI in the first hours after trauma [28]. Bhomia and colleagues identified 10 unique miRs (miR-151-5p, -195, -20a, -328, -362-3p, -30d, -451, -486, -505*, -92a in serum; miR-328, -362-3p, -451, -486 in CSF) to diagnose mild or moderate TBI and severe TBI [29]. Liu described the increased expression of miR-142-3p, -144, -340-5p, -674-5p, -153, -186, -190, -132*, -138-1*, and decreased let-7b expression in the hippocampus of rats with TBI compared to control [30]. Herrold’s review presents the results of an analysis of articles about known miRs as a biomarker for diagnosis, natural versus treatment-induced outcomes, and prognosis of TBI [31].

Despite a large number of publications devoted to the miR study in patients with TBI and various neurodegenerative diseases, some fundamental issues remain unclear in the practical application of miR because its kinetics and the influence of biological factors and identifying of the specific miRs is an extremely difficult task [32,33]. New methods of miR investigation in patients’ saliva significantly increased the rate of obtaining biological material in a non-invasive way thus illustrating the high potential of this direction in the practical field [34]. Moreover, experimental techniques are being developed in the field of therapeutics of miR-based drugs. At the moment, research is underway in the treatment of acute period of brain damage [35]. It is possible to expand the experience and accumulate information in problems of pDOC in future. Wen-Dong You et al. examined miR expression in cerebrospinal fluid patients who stayed unconscious for two weeks after TBI. The aim of the study was to detect differentially expressed miRs in the CSF of TBI patients remaining unconscious and to explore related single nucleotide polymorphisms. Authors have demonstrated that 14 miRs were differentially expressed, from which both upregulated miR-141, miR-257 and downregulated miR-1297 had the greatest fold-change [36]. Recent work presents data on an initial miR profile in pDOC patients. Authors identified differences in the expression of 48 miRs, the most interesting of which in terms of pDOC were miR-10b-5p, miR -335, -144, -151a, and -618, miR -335, -151a-3p, and miR -151a-5p. The last three showed correlation with neurobehavioral impairment. Patients with pDOC showed a different and reproducible miR expression pattern in comparison with controls. Future work on miR profiles will allow to provide the target therapy and patient-centric neurorehabilitation of pDOC [37].

The development of new diagnostic and prognostic tests in pDOC patients is relevant since the number of patients is increasing, doctors, healthcare organizers, and patients’ families need more accurate information about the prognosis and probable outcome of patients after severe brain damage. Electrophysiological and neuroimaging prognostic criteria are being developed, the specificity and sensitivity of which remains insufficient [38]. It is known that a single miR can regulate hundreds of targets, so we decided to test the expression of hsa-miR-21-5p, hsa-miR-93-5p, hsa-miR-191-5p, mmu-miR-499-5p, and hsa-let-7b-5p in patients with pDOC caused by TBI, SAH, and hypoxia. Since TBI is one of the main causes of pDOC, we selected miR whose levels have been previously associated with TBI, more specifically, for hsa-miR-93-5p, hsa-miR-191-5p, and mmu-miR-499-5p, whose levels were altered in TBI patients [23]. MiR 21 is a reliable biomarker for the diagnosis of severe TBI [39,40]. Let-7b was downregulated in the hippocampus of TBI rats compared to controls [30]. We wanted to compare the levels of these miRs in plasma and CSF of patients with pDOC to identify their correlation with the severity of pDOC. Obviously, miR levels in CSF reflect biochemical processes occurring in the brain. Since miRs pass through the blood–brain barrier, it is possible that they may also contribute to these same miR levels in the blood. In the present study, we analyzed the expression of the following miRs using qPCR: hsa-miR-21-5p, hsa-miR-93-5p, hsa-miR-191-5p, mmu-miR-499-5p, and hsa-let-7b-5p in CSF and plasma samples from a control group and patients with pDOC. 

## 2. Materials and Methods

### 2.1. Study Population

This study was performed according to Helsinki Declaration and was approved by the local ethics committee (№ 08-19/2 August 2019) of the Almazov National Medical Research Centre. All patients or patients’ representatives provided written informed consent. The analysis included 46 patients and 10 control patients. 

pDOC patients were treated in Polenov Neurosurgical Institute between January 2019 and December 2021. Inclusion criteria for pDOC patients were the following: age over 18 years, a history of TBI or non-TBI with an outcome in pDOC, and duration of the pDOC from 2 months up to 2 years. Exclusion criteria included somatic diseases and sepsis. 

During a neurological examination, before the final diagnosis of VS/UWS, MCS− or MCS+ patients were assessed by the CRS-R scale at least 5 times during the first week of admission, and the best result was selected for the group to determination. Depending on the results of the CRS-R assessment, patients were divided into 2 groups: group 1—VS/UWS, group 2—MCS− and MCS+ (Table 1). 

Together with researchers from the University of Liege from Coma Science Group, in order to overcome inaccuracies in the assessment of consciousness level and the differentiation between VS/UWS and MCS, the CRS-R index was calculated using the XL program jointly developed with Belgian scientists, which allows these calculations to be performed automatically (https://github.com/Annen/CRS-R/blob/master/CRS-R_index.R; date of accession: 16 February 2022). A CRS-R index value of 8.3 and below corresponded to the criteria of VS/UWS diagnosis and the indicator from 8.4 and above—to MCS− and MCS+. Detailed results of patients’ division according to the CRS-R scale and RS-R index are presented in Table 2. Group 1 included 18 patients in VS/MCS (average values of the CRS-R index–3.41); group 2 included 28 patients in MCS (11 MCS—patients and 17 MCS+ patients) (average values of the CRS-R index–26.41); the average age in each group was 42.1 and 35.2 years, correspondingly; the average duration of pDOC was 10.2 and 6.2 months.

All patients underwent blood sampling from the peripheral vein and lumbar puncture during the first week of hospitalization. Lumbar puncture and peripheral blood sampling were performed on the same day in the period from 9.00–10.00 am.

The control group included 10 patients operated for extracerebral volumetric neoplasms of the brain: meningiomas of various localizations, arterial aneurysm of the basilar artery. Inclusion criteria were the following: age over 30 years, and compensated somatic pathologies. Exclusion criteria included autoimmune diseases, central nervous system diseases, and sepsis. 

A sampling (of blood from a peripheral vein, and liquor through lumbar drainage) was performed immediately after induction anesthesia prior to surgical intervention. 

### 2.2. Sample Collection and Preparation

Whole blood was collected in Vacutainer tubes with EDTA (Vacutest, KIMA, Italy). CSF and venous blood samples were processed within 1.5 h after collection and kept at +4 °C during that time.

CSF and venous blood samples were centrifuged three times successively at 3000× *g* for 10 min at +4 °C using a swinging bucket rotor. After each centrifugation step, the supernatant was carefully transferred to a new conical tube. After the final centrifugation, the supernatant was aliquoted and stored at −80 °C for further use.

### 2.3. Isolation of Total RNA

Total RNA was isolated from CSF and plasma by phenol-chloroform extraction using the TRIzol LS reagent (Life Technologies Co., Carlsbad, CA, USA) as described in Kondratov et al. 2020 [41]. Briefly, 200 μL of CSF or plasma sample was mixed with 600 μL of TRIzol LS containing 1 μg of *Escherichia coli* tRNA (Sigma-Aldrich, St. Louis, MO, USA # R1753-100UN) and 10^8^ molecules of *Caenorhabditis elegans* synthetic oligoribonucleotide synth-cel-miR-39-3p (UCACCGGGUGUAAAUCAGCUUG) (Syntol, Moscow, Russia) identical to mature cel-miR-39-3p (miRbase: MIMAT000001). Synthetic oligoribonucleotide cel-miR-39-3p was used as an exogenous control to check the quality of miR isolation and to control the presence of inhibitors. GlycoBlue™ was used as coprecipitant (Thermo Fisher Scientific, Minneapolis, MN, USA). The dried RNA precipitate was dissolved in 12 μL of Rnase-free water (Ambion, Austin, TX, USA). The samples were stored in a low-temperature freezer at −80 °C. 

### 2.4. Reverse Transcription and qPCR

In this work expression levels of the following miRs were analyzed: hsa-miR-21-5p MIMAT0000076, hsa-miR-93-5p MIMAT0000093, hsa-miR-191-5p MIMAT0000440, mmu-miR-499-5p MIMAT0003482, and hsa-let- 7b-5p MIMAT0000063 (https://www.mirbase.org/; date of accession: 3 March 2022). Given that the total RNA was isolated from 200 μL of CSF or plasma and diluted in the same amount of water (12 μL), subsequent dilution was not required. 

TaqMan™ MicroRNA Reverse Transcription Kit (Thermo Fisher Scientific, Minneapolis, MN, USA) was used for reverse transcription according to manufacturer’s recommendations. The following TaqMan MicroRNA Assays were used to detect microRNA of interest: hsa-miR-191-5p (Assay ID 002299), hsa-miR-21-5p (Assay ID 000397), hsa-miR-93-5p (Assay ID 001090), mmu-miR-499-5p (Assay ID 001352), hsa-let-7b-5p (Assay ID 000378), and cel-miR-39 (Assay ID 000200) (Life Technologies, USA). Reverse transcription was performed in Veriti 96-Well Thermal cycler (Applied Biosystems, Waltham, MA, USA) with the following amplification program: 30 min at 16 °C, 30 min at 42 °C and 5 min at 85 °C. After reverse transcription, the cDNA was stored at −20 °C until the amplification. 

Amplification of miR targets was performed using TaqMan Universal Master Mix II no UNG (ThermoFisher Scientific, Waltham, MA, USA) and hydrolysis probes of TaqMan MicroRNA Assays (ThermoFisher Scientific, USA) according to manufacturer’s recommendations. MiR qPCR amplification was performed using LightCycler 480 II (Roche, Basel, Switzerland) with the following program: 95 °C for 10 min, followed by 40 cycles of 95 °C for 15 s and 60 °C for 1 min. Calibration curves were constructed using a series of 10-fold dilutions of synthetic RNA oligonucleotides (Syntol, Moscow, Russia), identical to mature target miRs.

### 2.5. qPCR Data Analysis

Raw data from thermocycler software were analyzed using GraphPad Prism 5 software (San Diego, CA, USA). MiR Cq data were normalized by external synthetic oligoribonucleotide cel-miR-39-3p. Each normalized Cq was calculated as described in Kondratov et al., 2020 [41]. Briefly, the following formula was used: CqmiR_norm = CqmiR-(CqmiR-39-CqmiR-39_median), where CqmiR is the target miR Cq in the sample, CqmiR-39 is miR-39-3p Cq in this sample, and CqmiR-39_median is median miR-39-3p Cq. The statistical Mann–Whitney U-test was used to assess differences between the two groups.

### 2.6. MiR–Disease Association Network Analysis

The miR–severe brain damage association network analysis was performed using web-based tool miRNet 2.0 (https://www.mirnet.ca; date of accession: 3 October 2022).

## 3. Results

**Figure 1:**
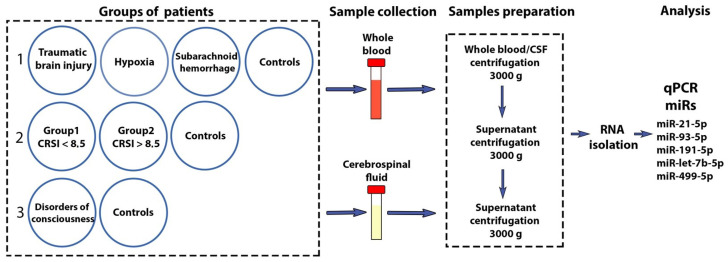
Study design. There are three types of patient division into groups: 1—division according to etiology, 2—division based on the CRS-R index, 3—total patients group. qPCR—quantitative polymerase chain reaction.

The expression levels of miR-21-5p, miR-93-5p, miR-191-5p, miR-499-5p, and let-7b-5p in CSF and plasma samples in groups of patients with disorders of consciousness and the control group were analyzed in the present study (Figure 1). 

### 3.1. Description of Patient Groups

The analysis included 46 patients and 10 control patients. We compared the total group of patients with pDOC and the control group; moreover, patients with vegetative state were divided into subgroups in two ways: according to the etiology of the disease and by CRS-R index (Table 1 and Table 2). Based on the etiology of the disease, patients were divided into three groups: with TBI (22 patients), hypoxia (17 patients), and SAH (7 patients) (Table 1). Based on the CRS-R index, patients were divided into two groups: group 1 included 18 patients in VS/MCS; group 2 included 28 patients in MCS (11 MCS− patients and 17 MCS+ patients) (Table 2).

### 3.2. MiR Expression Levels

**Figure 2:**
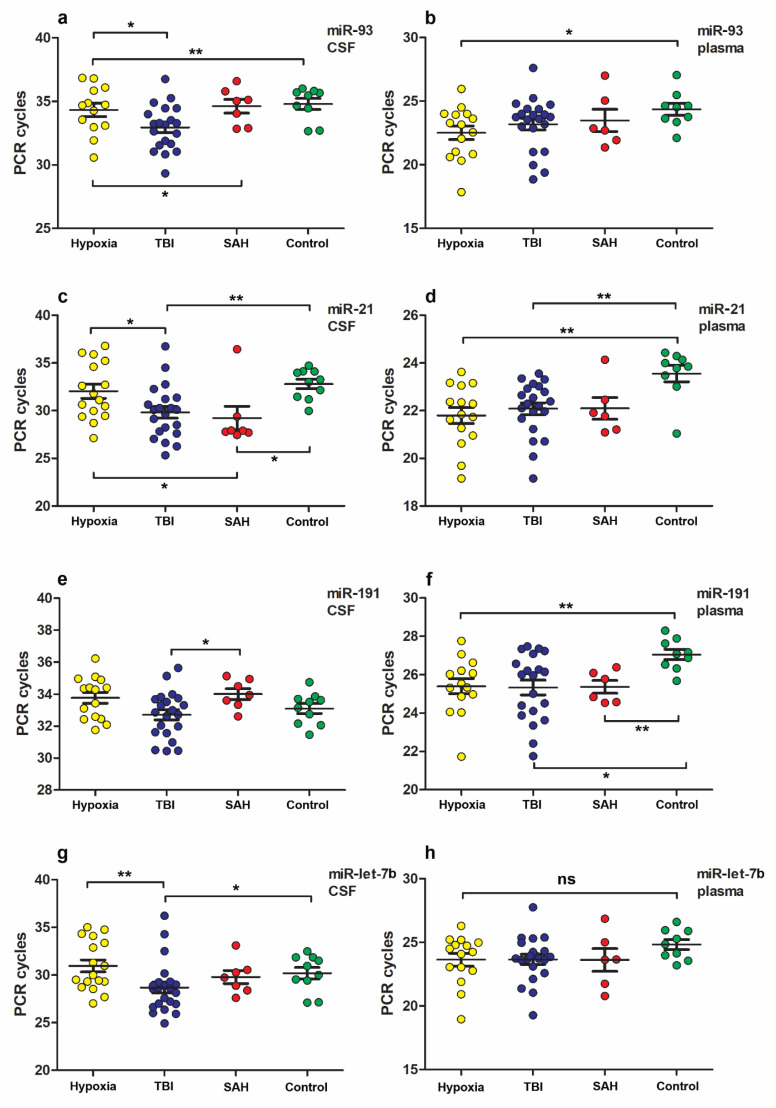
MiRs expression levels between the groups of patients divided according to etiology of the disease and the control group. (**a**,**b**) Increased miR-93 expression levels in CSF (**a**) and plasma (**b**) samples; (**c**,**d**) increased miR-21 expression levels in CSF (**c**) and plasma (**d**) samples; (**e**,**f**) increased miR-191 expression levels in CSF (**e**) and plasma (**f**) samples; (**g**,**h**) increased miR let-7 expression levels in CSF samples, but not in plasma samples (**h**). U-Mann–Whitney test was used to assess differences between the means of two groups. TBI (*n* = 22 patients), hypoxia (*n* = 17 patients), SAH (*n* = 7 patients), control (*n* = 10 patients). One asterisk, *p* < 0.05; two asterisks *p* < 0.01; ns, *p* > 0.05.

The levels of miR in the patient group divided according to the etiology of the disease are shown on Figure 2. As a result of our study, we demonstrated an increase in levels of miR-93, -21, -191, let-7b, but not miR-499 in the patient groups compared to the control group. 

The most significant differences in the levels of miR-93 between groups of patients, divided by the etiology of the disease, were found in CSF samples between the groups: TBI vs. hypoxia, TBI vs. SAH, and TBI vs. control (Figure 2a, Table 3), as well as in plasma samples of hypoxia vs. control group (Figure 2b).

Notable differences in miR-21 levels were found in CSF samples between groups: TBI vs. hypoxia, hypoxia vs. SAH, TBI vs. control, SAH vs. control (Figure 2c, Table 3), as well as in plasma samples in groups: TBI vs. control and hypoxia vs. control (Figure 2d).

Differences in miR-191 levels were found in CSF samples only between groups TBI vs. SAH (Figure 2e, Table 3), as well as in plasma samples in groups: TBI vs. control, hypoxia vs. control, SAH vs. control (Figure 2f).

Differences in miR-let-7b levels were found only in CSF samples between groups: TBI vs. hypoxia and TBI vs. control (Figure 2g, Table 3), but not in plasma samples (Figure 2h).

However, there were found no differences in the level of miR-499 either in CSF or plasma samples in compared groups.

The only differences in miR levels found both in CSF and plasma samples were in the TBI vs. control group only for miR-21 (Figure 2c,d, Table 3). 

**Figure 3:**
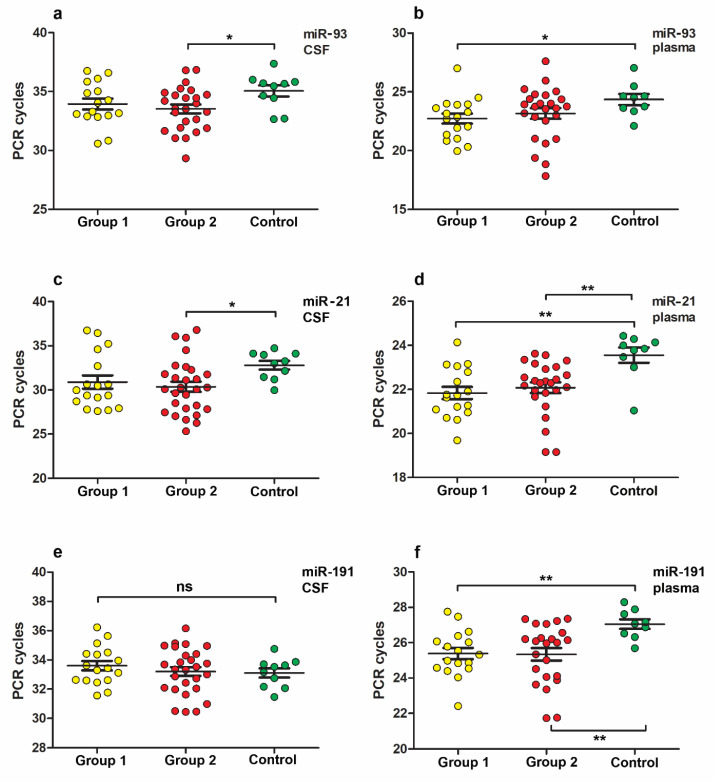
MiRs expression levels between two groups of patients divided according to CRS-R index and the control group. (**a**,**b**) Increased miR-93 expression levels in plasma (**a**) and CSF (**b**) samples of group 2 vs. control and group 1 vs. control; (**c**,**d**) increased miR-21 expression levels in plasma (**c**) and CSF (**d**) samples of group 2 vs. control, group 1 and 2 vs. control; (**e**,**f**) increased miR-191 expression level in plasma samples (**f**) group 1 and 2 vs. control, but not in CSF samples (**e**). U-Mann–Whitney test was used to assess differences between the means of two groups. Group 1 VS/MCS (*n* = 18 patients), group 2 MCS (*n* = 28 patients), control (*n* = 10). One asterisk, *p* < 0.05; two asterisks *p* < 0.01; ns, *p* > 0.05.

In the case of separating the pDOC patient group by CRS-R, an increase in miR levels was observed only for miRs-93, -21, -191, but not for miR-let-7b and -499 (Figure 3). Increased levels of miR-93 were identified in group 2 vs. control in CSF samples and in group 1 vs. control in plasma samples (Figure 3a,b).

Additionally, miR-21 level in group 2 vs. control was increased in CSF samples and in groups 1 and 2 vs. control in plasma samples (Figure 3c,d). 

It is noteworthy that expression levels of miR-191 showed a significant increase only in plasma samples in groups 1 and 2 vs. control (Figure 3f) but not in CSF samples (Figure 3e). 

No statistically significant differences were found between the expression levels of miR-499 and let-7b in the CSF or plasma samples in groups of patients with pDOC divided according to CRS-R index and the control group (Table 4). 

**Figure 4:**
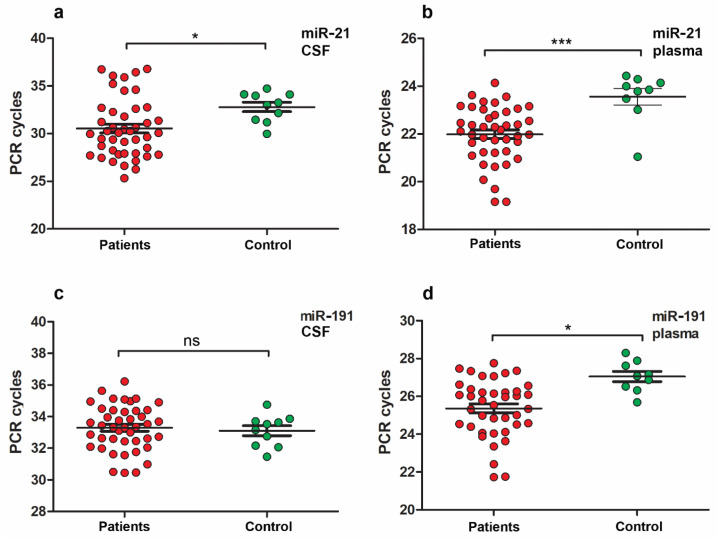
MiR expression levels in patient’s group with pDOC and the control group. (**a**,**b**) MiR-21 expression levels in CSF (**a**) and plasma (**b**) samples. (**c**,**d**) MiR-191 expression levels in CSF (**c**) and plasma (**d**) samples. The total group of patients with pDOC (*n* = 46), control (*n* = 10). One asterisk, *p* < 0.05; three asterisks, *p* < 0.001; ns, *p* > 0.05.

In the entire group of patients with pDOC, a significant increase in miR-21 expression level was found between all patients and the control group in CSF (Figure 4a) and plasma samples (Figure 4b). 

Apart from that, an increase in miR-191 expression level was found in plasma samples between all patients and the control group (Figure 4d) but not in CSF samples (Figure 4c).

No statistically significant differences were found between the miR expression levels of miR-93, -499, -let-7b in the CSF or plasma samples in the entire group of patients with pDOC compared to the control group. 

Finally, we observed the greatest differences in miR levels compared to controls when separating patients with pDOC according to the cause of the disease (miR-93,-191, -21, -let-7). At the same time, we obtained the smallest differences in the types of altered miRs when we analyzed the pooled groups with pDOC compared to controls (miR-21, -191).

## 4. Discussion

Interest in miR molecules as potential biomarkers of various diseases has significantly increased in recent decades [17,42,43,44]. The idea of using these molecules as potential diagnostic markers is based on their biology. In particular, these molecules are known to be involved in posttranslational regulation of the expression of up to 50% of genes [45,46]. Their levels can change rapidly, for example, in response to changes in the human physiological state or the onset of disease [47]. MiR levels change in the first stages of disease formation, when clinical symptoms are not yet evident. Early prediction of disease progression allows to start treatment earlier or to change the treatment tactics and to achieve a significant improvement in the patient’s quality of life. 

In our study, we intended to find miRs associated with different causes leading to pDOC in order to differentiate patient groups in terms of future prognosis and survival. Our results indicate that in pDOC patients divided into groups according to the etiology of the disease, levels of miR-93, -21, and -191 in CSF and plasma samples were increased in some patient groups (Figure 2). Marked differences in miR-let-7 levels were found only in CSF samples but not in plasma. At the same time, no differences in miR-499 levels were found in either CSF or plasma samples in the compared groups.

It is known that miR-93, miR-191, and miR-499 miR levels were elevated in the serum of patients with mild, moderate, and severe trauma [23]. Our results corroborate those of Yang and colleagues, namely, we also found elevated levels of miR-93 and -191 in the group of patients with TBI compared with the control group. In our study, however, we found no change in miR-499 levels. This may be due to the small sample size in our study, as we included 22 TBI patients and 10 controls, whereas Young’s analysis included 76 TBI patients and 38 controls. In addition, the difference in results may depend on the timing of material collection from the patient after TBI. 

Moreover, we found increased miR-21 levels in plasma and CSF samples from patients with TBI and in plasma samples from patients with hypoxia compared with controls, which is consistent with Di Pietro et al.’s data where miR-21 was significantly elevated in TBI and was a reliable biomarker for its diagnosis [39].

We also found differences in the levels of miR-93, -21, and let-7b in CSF samples between groups of patients with TBI and hypoxia (Figure 2). This difference was not detected in plasma samples and could probably be related to abnormal brain activity. Further studies of the expression levels of these miRs in patients with TBI and hypoxia are of interest. Differences between groups of patients with TBI, hypoxia and SAH are difficult to explain, since the exact mechanisms underlying these effects are currently unknown. 

The aim of our study was also to test whether the miRs we analyzed could be used to differentiate patients, including CRSI groups. Among the analyzed miRs, we did not find a marker that would help to distinguish VS/UWS patient groups from MCS. However, the investigated groups differed significantly from the controls in the levels of miRs-93, -21, and -191.

Analyzing miR-191 expression levels in patient groups divided according to different criteria (etiology, CRS-R index, pooled group), a marked increase in levels was detected in plasma samples, but not in CSF. It is likely that these differences are not related to impaired brain activity or impaired consciousness, but possibly to a state of prolonged hypodynamia in patients. 

In the case of analysis of miR levels between the pooled patient group and controls, the only found difference was in miR-21 levels in plasma and CSF samples (Figure 4).

**Figure 5:**
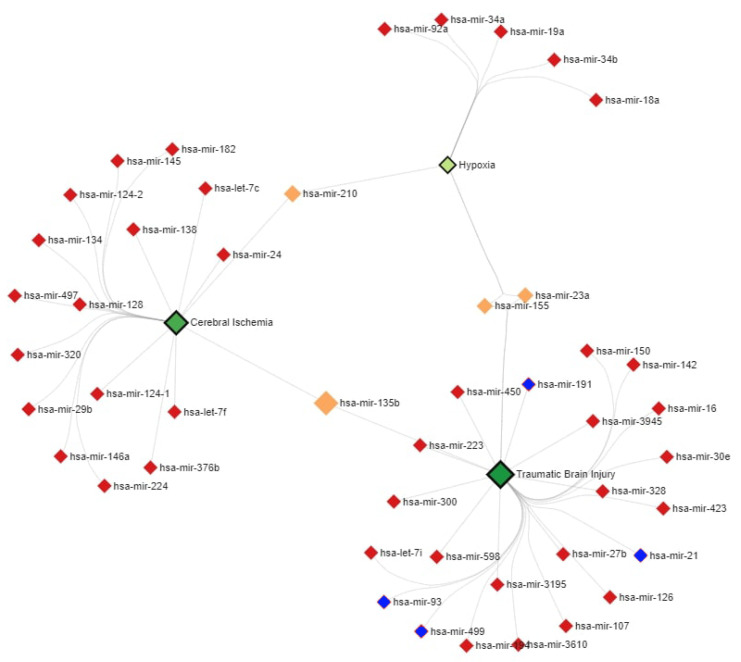
MiR–severe brain damage association network analysis. Green diamonds show variants of severe brain damage (TBI, hypoxia, cerebral ischemia). Red diamonds depict miRs associated with severe brain damage. Orange diamonds mark miRs common for TBI, hypoxia, and cerebral ischemia. Blue diamonds depict miRs taken into analysis. The analysis was performed using web-based tool miRNet 2.0.

Using the mirNet tool, we identified the miRs whose levels were altered in pDOC diseases (Figure 5). Four of the five miRs we studied were represented in the association network of TBI. 

It should be emphasized that we specifically used two tissue types (plasma and CSF) in the analysis to see which one would be more informative for further diagnosis. We also thought that if we could detect common biomarkers for both plasma and CSF, we would use the least invasive method of material collection—plasma. We showed that there were a number of miRs that changed only in CSF samples, several miRs altered only in plasma samples, and there were miRs that changed in plasma and liquor samples. Namely, we found that miR-93 was elevated in the group of patients with hypoxia compared to controls in both plasma and CSF. Moreover, miR-21 was elevated in TBI group in both plasma and CSF (Figure 2a–d).

pDOC is a complex disease that can be caused by a variety of reasons. Dividing patients into groups according to CSRI is based on a comprehensive check for the presence of consciousness. This division does not consider the cause of pDOC, and consequently does not take into account which molecular mechanism is impaired. However, at the molecular level, microRNA expression is strongly dependent on the cause of pDOC (TBI, hypoxia, SAH), at least at the beginning of the disease. Therefore, when analyzing microRNA levels as a prognostic marker in patients with pDOC, the starting point seems to be the cause that led to the development of the disease. We can use the investigated markers to confirm the disease in pDOC patients.

There are no extensive studies in the literature on miR levels in pDOC. The present study serves as a small step toward understanding the mechanisms of dysregulation through the miR system in pDOC. Despite the large number of publications devoted to the study of miRs in patients with TBI and various neurodegenerative diseases, some fundamental questions about the practical application of miRs remain unclear, such as their kinetics and the influence of biological factors, as well as the identification of specific miRs being extremely challenging [32,33]. Further experience and accumulation of information on pDOC issues is possible in the future. 

This study has several limitations. First, the number of samples in the group of patients with SAH was limited. Second, the control group was not composed of healthy volunteers; it was a group of patients with extracerebral volumetric neoplasms of the brain without central nervous system diseases. Third, we analyzed a limited set of miRs.

## Figures and Tables

**Table 1:** Results of patient division according to the CRS-R scale and RS-R index. Distribution by etiology, male, and female in each group.

		Group				Total	
		1		2			
		N	%	N	%	N	%
Etiology (TBI, hypoxia, SAH)	Hypoxia	9	50.0%	8	28.6%	17	37.0%
	SAH	5	27.8%	2	7.1%	7	15.2%
	TBI	4	22.2%	18	64.3%	22	47.8%
Total		18	100.0%	28	100.0%	46	100.0%
Gender	female	7	38.9%	10	35.7%	17	37.0%
	male	11	61.1%	18	64.3%	29	63.0%
Total		18	100.0%	28	100.0%	46	100.0%

**Table 2:** Detailed results of patient division according to the CRS-R scale and RS-R index.

	Group	N	Average	Median	Min	Max	Q1 (25%)	Q3 (75%)
Duration of pDOC	1	18	10.2	3.5	1.0	54.0	1.0	11.0
	2	28	6.2	2.5	1.0	37.0	1.0	5.5
Age	1	18	42.1	44.0	18.0	63.0	33.0	54.0
	2	28	35.2	32.5	21.0	61.0	25.0	44.0
CRS-R Score—at admission	1	18	4.4	4.5	2.0	6.0	4.0	5.0
	2	28	9.8	9.0	6.0	15.0	7.0	12.0
CRS-R Score after one month	1	18	5.7	5.0	3.0	23.0	4.0	6.0
	2	28	12.0	12.0	5.0	23.0	8.0	15.0
CRS-R index at admission	1	18	3.41	3.80	0.33	4.84	2.75	4.50
	2	28	26.41	20.80	12.13	64.57	14.22	39.56
CRS-R index after one month	1	18	9.10	3.80	2.41	100.00	2.75	4.84
	2	28	37.67	32.79	3.80	99.67	14.74	52.58

**Table 3:** MiR expression levels in CSF and plasma samples. Patient division according to the etiology of the disease.

Groups by Etiology CSF/Plasma	*p*-Value miR-93	*p*-Value miR-21	*p*-Value miR-191	*p*-Value miR-let-7b	*p*-Value miR-499
**TBI vs. Hypoxia**	**0.0487**/0.3357	**0.0357**/0.5004	0.0542/0.9442	**0.0034**/0.7850	0.1193/0.6303
**TBI vs. SAH**	**0.0434**/0.8383	0.3396/0.5794	**0.0411**/0.9273	0.1201/0.7484	0.2515/0.9071
**Hypoxia vs. SAH**	0.9368/0.5593	**0.0252**/0.7853	0.5258/0.7728	0.3739/0.8457	0.7268/0.5333
**TBI vs. Control**	**0.0083**/0.1606	**00038/0.0013**	0.4220/**0.0133**	**0.0328**/0.0855	0.6113/0.6837
**Hypoxia vs. Control**	0.8412/**0.0235**	0.3990/**0.0024**	0.2330/**0.0042**	0.7632/0.1360	0.5468/0.3553
**SAH vs. Control**	0.9182/0.2721	**0.0136**/0.0768	0.1088/**0.0048**	0.6691/0.3277	0.7396/0.9061

**Table 4:** MiR expression levels in CSF and plasma samples. Patient division according to CRS-R Index.

CSF/Plasma	*p*-Value miR-93	*p*-Value miR-21	*p*-Value miR-191	*p*-Value miR-let-7	*p*-Value miR-499
**Group 1vs. Group 2**	0.6400/0.2334	0.7343/0.2334	0.5230/0.8481	0.7180/0.6912	0.3141/0.4344
**Group 1 vs. Control**	0.2799/**0.0132**	0.0564/**0.0033**	0.4666/**0.0030**	0.6488/0.0948	0.5814/0.3186
**Group 2 vs. Control**	**0.0214**/0.1977	0.0148/**0.0012**	0.7582/**0.0057**	0.1982/0.1095	0.8976/0.7696

## Data Availability

Not applicable.
